# Index tracking strategy based on mixed-frequency financial data

**DOI:** 10.1371/journal.pone.0249665

**Published:** 2021-04-06

**Authors:** Xiangyu Cui, Xuan Zhang

**Affiliations:** 1 School of Statistics and Management, Shanghai University of Finance and Economics, Shanghai, China; 2 Shanghai Institute of International Finance and Economics, Shanghai University of Finance and Economics, Shanghai, China; The Bucharest University of Economic Studies, ROMANIA

## Abstract

To obtain market average return, investment managers need to construct index tracking portfolio to replicate target index. Currently, most literatures use financial data that has homogenous frequency when constructing the index tracking portfolio. To make up for this limitation, we propose a methodology based on mixed-frequency financial data, called FACTOR-MIDAS-POET model. The proposed model can utilize the intraday return data, daily risk factors data and monthly or quarterly macro economy data, simultaneously. Meanwhile, the out-of-sample analysis demonstrates that our model can improve the tracking accuracy.

## Introduction

The index tracking strategy, which aim at tracking the return of a given stock index when constructing the portfolio, is a major strategy adopted by fund managers. [1–7] theoretically and experimentally study the index tracking strategy under different constraints in reality, e.g. the number of stocks in the portfolio is limited.

The simplest and most widely used index tracking strategy is the global minimum variance strategy. Let ***R***_*t*_ be the vector of daily excess returns of stocks over the target index and ***ω***_*t*_ be the global minimum variance portfolio weights. The difference between return of the index tracking strategy and return of the target index is $\omega_{t}^{T}R_{t}$ on day *t*. Mathematically, the investors aim to minimize tracking error, which is measured by variance of the difference between return of the index tracking strategy and return of the target index, i.e.,

$$\begin{array}{c} \underset{\omega_{t}}{\text{min}}\;\omega_{t}^{T}\Sigma_{t}\omega_{t}\;\text{s}.\text{t}.\;\omega_{t}^{T}1=1, \end{array}$$

where **Σ**_*t*_ is the *N* × *N* conditional covariance matrix of ***R***_*t*_. Obviously, the optimal index tracking strategy is

$$\begin{array}{c} \omega_{t}^{min}=\frac{\Sigma_{t}^{-1}1}{1^{T}\Sigma_{t}^{-1}1}. \end{array}$$

We can see that the key part of global minimum variance strategy is to estimate the covariance matrix or inverse covariance matrix.

In the literature, methods for estimating the covariance matrix or inverse covariance matrix mainly focus on financial data with homogenous frequency. [8–10] try to estimate the covariance matrix based on quarterly, monthly and daily returns, respectively. [5, 11–18] aim to improve covariance matrix estimation using intraday data. Differently [19–23], focus on the estimation of inverse covariance matrix. With the improvement in high-speed computation and large amounts of storage, financial data streams become more and more real-time and complex, such as high-frequency data and ultra-high-frequency data. Besides the historical data in financial markets, monthly or quarterly macro-economic factors are also valuable information sources of stocks’ volatilities (see [24–29] further reveal that macro-economic factors, such as GDP growth, exchange rate and short-term interest rate, are important explanatory variables of the slow-moving component in volatilities.

However, due to the heterogeneous frequency of macro-economic factors and historical data, it is a great challenge to construct a unified econometric model. Among all proposed models, the mixed data sampling (MIDAS) method in [30] attracts great attentions, and induces several important extensions, such as GARCH mixed-data sampling (GARCH-MIDAS) model (see [31]), Factor-based mixed-data sampling (Factor-MIDAS) model (see [32]). Different from the MIDAS method, other economic models handling mix-frequency data, such as High-frequency-based volatility (HEAVY) model (see [33]); Factor GARCH-Itô model (see [34]), are mainly focusing on integrating the intraday financial data and daily financial data. Compared with homogenous-frequency models, mixed-frequency model contains more information in the original data, which can better capture market and have better accuracy in prediction. It provides a timely update on portfolio and helps fund managers achieve targeted index tracking performance.

In this paper, we propose a general framework for mixed-frequency financial data, called FACTOR-MIDAS-POET model, to estimate the covariance matrix. The proposed model combines monthly macro-economic factors, daily observable factors (market return and the innovation of VIX) and intraday returns to improve covariance matrix estimation. In empirical analysis, we compare our model with existing models in the literature, and find that the tracking accuracy of minimum variance tracking strategy is greatly improved by using the proposed model. The reason is that compared with other models, our model contains macro-economic information and option market information. We also find that when the amount of historical data decreases, performances of the index tracking portfolios based on different models all decrease, but our model is less affected. Moreover, when the estimation window is short (e.g., 3 months), integrating intraday return data into our model may yield better tracking performance than not using the intraday return data.

The remaining paper is organized as follows. In the estimations of the covariance and its inverse, we introduce existing methods for estimating the covariance matrix and inverse covariance matrix based on homogenous frequency data. In FACTOR-MIDAS-POET method and estimation, we introduce the FACTOR-MIDAS-POET model and its estimation method. In data and descriptive analysis, we explain the data used in this paper. In empirical study, we conduct the empirical study and compare performances of different models. In conclusions and discussion, we conclude our paper.

## The estimations of the covariance and its inverse

To obtain the minimum variance index tracking strategy, there are two main ways. The first one is to estimate the covariance matrix and then obtain the inverse matrix; The second one is to estimate the inverse covariance matrix directly. The rest of this section summarizes the important existing methods for estimating the covariance matrix and inverse covariance matrix.

### Estimators of covariance matrix

Based on the daily financial data, we summarize three estimators as follows,

$$S_{t,1}=\frac{1}{T-1}\sum_{k=1}^{T}\left(R_{t-k}-\bar{R}\right){\left(R_{t-k}-\bar{R}\right)}^{T},$$ (1)

$$\begin{array}{c} S_{t,2}=S_{t,1}+\frac{1}{T}\sum_{\ell =1}^{L}\sum_{k=1}^{T-\ell }(1-\frac{\ell }{L+1})[\left(R_{t-k-\ell }-\bar{R}\right){\left(R_{t-k}-\bar{R}\right)}^{T}\\ \;+\left(R_{t-k}-\bar{R}\right){\left(R_{t-k-\ell }-\bar{R}\right)}^{T}], \end{array}$$ (2)

$$S_{t,3}=\text{exp}\left(-\alpha \right)S_{t,1}+\alpha \;\text{exp}\left(-\alpha \right)\left(R_{t-1}-\bar{R}\right){\left(R_{t-1}-\bar{R}\right)}^{T}.$$ (3)

***S***_*t*,1_ is the sample covariance matrix. ***S***_*t*,2_ is the weighted lead and lag covariance matrix proposed by [35], which is designed to eliminate the non-synchronous trading effect. In this paper, *L* is set to 3 for daily return following [36]. ***S***_*t*,3_ is the backward-looking rolling estimator proposed by [10].

When we have intraday financial data, these estimators are modified as follows,

$$S_{t,1}=\frac{1}{T-1}\sum_{k=1}^{T}\sum_{i=1}^{M}R_{i,t-k}R_{i,t-k}^{T},$$ (4)

$$S_{t,2}=S_{t,1}+\frac{1}{T}\sum_{k=1}^{T}\sum_{\ell =1}^{L}\sum_{i=\ell +1}^{M}\left(R_{i-\ell ,t-k}R_{i,t-k}^{T}+R_{i,t-k}R_{i-\ell ,t-k}^{T}\right),$$ (5)

$$S_{t,3}=\text{exp}\left(-\alpha \right)S_{t,1}+\alpha \;\text{exp}\left(-\alpha \right)\sum_{i=1}^{M}R_{i,t-1}R_{i,t-1}^{T},$$ (6)

where ***R***_*i*,*t*−*k*_ is the vector of excess returns at time *i* in day *t* − *k*. ***S***_*t*,1_ is proposed by [12]. ***S***_*t*,2_ is proposed by [5]. If overnight returns are involved in the estimators, we have

$$S_{t,1}=\frac{1}{T-1}\sum_{k=1}^{T}(\sum_{i=1}^{M}R_{i,t-k}R_{i,t-k}^{T}+\left(R_{0,t-k}-{\bar{R}}_{0}\right){\left(R_{0,t-k}-{\bar{R}}_{0}\right)}^{T}),$$ (7)

$$S_{t,3}=\text{exp}\left(-\alpha \right)S_{t,1}+\alpha \;\text{exp}\left(-\alpha \right)(\sum_{i=1}^{M}R_{i,t-1}R_{i,t-1}^{T}+\left(R_{0,t-1}-{\bar{R}}_{0}\right){\left(R_{0,t-1}-{\bar{R}}_{0}\right)}^{T}),$$ (8)

where ***R***_0,*t*−*k*_ is the overnight return in day *t* − *k*.

### Estimators of inverse covariance matrix

The estimators of inverse covariance matrix are often built upon the estimators of covariance matrix. We summarize these estimators in this subsection.

$$S_{t,4}^{inv}=\left(T-N-1\right)S_{t,1}^{-1},$$ (9)

$$S_{t,5}^{inv}=\alpha \left(T-N-1\right)S_{t,1}^{-1}+\alpha \frac{N^{2}+N-2}{tr(S_{t,1})}I+\left(1-\alpha \right)\frac{NT-2}{tr(S_{t,1})}I,$$ (10)

$$S_{t,6}^{inv}=\left(T-N-1\right)S_{t,1}^{-1}+\frac{N^{2}+N-2}{tr(S_{t,1})}\frac{u}{2}I,$$ (11)

$$S_{t,7}^{inv}=\left(T-N-1\right)\left(1-u^{\frac{1}{2}}\right)S_{t,1}^{-1}+\frac{(T-N-1)N}{tr(S_{t,1})}u^{\frac{1}{2}}I,$$ (12)

$$S_{t,8}^{inv}=\left(T-N-1\right)S_{t,1}^{-1}+\frac{2(N-1)}{tr(S_{t,1}^{2})}S_{t,1},$$ (13)

$$S_{t,9}^{inv}=HL^{-1}CH^{T},$$ (14)

where *tr*(⋅) is the trace of a matrix, $u=Ndet{\left(S_{t,1}\right)}^{\frac{1}{N}}tr{\left(S_{t,1}\right)}^{-1}$, *det*(⋅) is the determinant of a matrix, ***H*** is an orthogonal matrix and ***L*** is a diagonal matrix such that ***S***_*t*,1_ = ***HLH***^*T*^, ***C*** is a diagonal matrix with elements *c*_*i*_ = *T* + *N* − 2*i* − 1 for *i* = 1, …, *N*. $S_{t,4}^{inv}$ is proposed by [19]. $S_{t,5}^{inv}$ is the shrinkage estimator proposed by [19]. $S_{t,6}^{inv}$ is proposed by [20]. $S_{t,7}^{inv}$ is proposed by [21]. $S_{t,8}^{inv}$ is proposed by [22], $S_{t,9}^{inv}$ is proposed by [23].

## FACTOR-MIDAS-POET method and estimation

There are two restrictions of the methods summarized in Section 2. The first one is that these classical methods require a homogenous sampling frequency, leading to a low usage of information contained in mixed-frequency financial data; The second is that these classical methods do not take monthly or quarterly macro-economic factors into consideration. To remedy these limitations, we propose a model involving multi-frequency financial data to better reflect financial market.

In our model, excess returns are driven by both observable and unobservable factors. Meanwhile, excess returns are influenced by the status of macro economy. More specifically, the model is shown as follows,

$$r_{i,t,m}=a^{T}\tau_{m}F_{obs,t,m}+b^{T}F_{unobs,t,m}+\epsilon_{i,t,m},$$ (15)

$$\tau_{m}=\beta_{0}+\beta^{T}\sum_{k=1}^{K}B\left(k,\theta \right)X_{m-k},$$ (16)

$$B(k,\theta )=\frac{{\left(\frac{k}{K}\right)}^{\theta_{1}-1}{\left(1-\frac{k}{K}\right)}^{\theta_{2}-1}}{\sum_{k=1}^{K}{\left(\frac{k}{K}\right)}^{\theta_{1}-1}{\left(1-\frac{k}{K}\right)}^{\theta_{2}-1}},$$ (17)

where ***r***_*i*,*t*,*m*_ is the vector of excess returns of *N* stocks at time *i* in day *t* and month *m*, ***F***_*obs*,*t*,*m*_ is the vector of daily observable factors in day *t* and month *m*, ***F***_*unobs*,*t*,*m*_ is the vector of daily unobservable factors in day *t* and month *m*, ***ϵ***_*i*,*t*,*m*_ is the *N*-dimensional residual vector, *τ*_*m*_ is the long-run component associated with monthly observable macro-economic factors, ***X***_*k*_ is the vector of the macro-economic factors in month *k*, *B*(*k*, ***θ***) is the widely used Beta weighted lag structure in MIDAS model. We need to mention that *τ*_*m*_ is known for any day *t* in month *m*, and is updated in the beginning of the next month *m* + 1.

Based on principal orthogonal complement thresholding(POET) method in [37], the daily covariance matrix of excess returns, ${\hat{\Sigma }}_{t,m,}$, is estimated as follows,

$${\hat{\Sigma }}_{t,m}=\tau_{m}^{2}a^{T}{\hat{\Sigma }}_{t,m,obs}a+{\hat{R}}_{J}+{\hat{R}}_{N-J},$$ (18)

where ${\hat{R}}_{J}=\sum_{j=1}^{J}{\hat{\lambda }}_{j}{\hat{u}}_{j}{\hat{u}}_{j}^{T}$, ${\hat{R}}_{N-J}=\sum_{k=J+1}^{N}{\hat{\lambda }}_{k}{\hat{u}}_{k}{\hat{u}}_{k}^{T}={\left({\hat{r}}_{ij}\right)}_{N\times N}$, ${\hat{\lambda }}_{1}\geq \ldots ,\geq {\hat{\lambda }}_{n}$ are the eigenvalues of the covariance matrix of ***r***_*i*,*t*,*m*_ − ***a***^*T*^ *τ*_*m*_ ***F***_*obs*,*t*,*m*_, ${\hat{u}}_{j}$ is the eigenvector associated with ${\hat{\lambda }}_{j}$. It is unrealistic to assume that the matrix ${\hat{R}}_{J}$ is sparse because of the existence of common factors. But it is reasonable to assume that the matrix ${\hat{R}}_{N-J}$ is sparse. To guarantee sparsity, it is natural to set a threshold to shrink the non-diagonal elements of ${\hat{R}}_{N-J}$ into

$$\begin{array}{c} {\tilde{R}}_{N-J}={\left({\hat{r}}_{ij}^{T}\right)}_{N\times N},\;{\hat{r}}_{ij}^{T}=\{\begin{array}{c} {\hat{r}}_{ii},i=j\\ s\left({\hat{r}}_{ij}\right)I\left(|{\hat{r}}_{ij}|\geq \tau \right),i\neq j \end{array}, \end{array}$$ (19)

where *s*(⋅) is a generalized shrinkage function, *τ* is the threshold, *I*(⋅) is the indicator function. Then, we obtain the FACTOR-MIDAS-POET estimator of the covariance matrix as follows,

$${\hat{\Sigma }}_{t,m,FMP}=\tau_{m}^{2}a^{T}{\hat{\Sigma }}_{t,m,obs}a+{\hat{R}}_{J}+{\tilde{R}}_{N-J}.$$ (20)

Based on [38], we apply the method of linear compression to obtain the shrinkage inverse covariance matrix estimator as follows:

$$S_{t,m,FMP}^{inv}=c_{1}{\left(S_{t,1}\right)}^{-1}+c_{2}I+c_{3}{\hat{\Sigma }}_{t,m,FMP}^{-1},$$ (21)

where *c*_1_, *c*_2_ and *c*_3_ are combination coefficients, ${\hat{\Sigma }}_{t,m,FMP}$ is the target matrix. The optimized solution and expression can be found in [38].

## Data and descriptive analysis

### Data

In empirical analysis, we use the stocks listed in Dow Jones Industrial Average (DJIA) to track the S&P 500 index. The stock tickers and full company names of 30 stocks listed in DJIA are available in Table 1. As there are too many missing values in the TAQ data files of TRV company, we remove this company and use the rest 29 DJIA stocks to track S&P 500 index. The sample period in our analysis is from Jan. 1st, 2006 to Dec. 31st, 2011. The daily and 5 minutes (5-min) data of S&P 500 index are obtained from Tick Data. The 5-min data of 30 DJIA stocks are collected from NYSE Trade and Quotations (TAQ) database. Considering the higher possibility of including biases and reporting errors in the first 30 minutes after opening, we discard the first 30 minutes data. Thus, there are 72 intraday 5-min returns and one overnight return in each trading day.

**Table 1:** Tickers and company full name of DJIA component stocks on June 8, 2009.

Ticker	Company Name
AA	Alcoa Inc.
AXP	American Express Company
BA	The Boeing Company
BAC	Bank of America Corporation
CAT	Caterpillar Inc.
CSCO	Cisco Systems, Inc.
CVX	Chevron Corporation
DD	E.I. du Pont de Nemours & Company
DIS	The Walt Disney Company
GE	General Electric Company
HD	The Home Depot, Inc.
HPQ	Hewlett-Packard Company
IBM	International Business Machines Corporation
INTC	Intel Corporation
JNJ	Johnson & Johnson
JPM	JPMorgan Chase & Co.
KFT	Kraft Foods Inc.
KO	The Coca-Cola Company
MCD	McDonald’s Corporation
MMM	3M Company
MRK	Merck & Co., Inc.
MSFT	Microsoft Corporation
PFE	Pfizer Inc.
PG	The Procter & Gamble Company
T	AT&T Inc.
TRV	The Travelers Companies, Inc.
UTX	United Technologies Corporation
VZ	Verizon Communications Inc.
WMT	Wal-Mart Stores, Inc.
XOM	Exxon Mobil Corporation

The daily value-weighted market return and daily VIX are considered as daily observable factors and obtained from CRSP database and Chicago Board Options Exchange (CBOE), respectively.

The monthly macro-economic factors includes eight important ones:

1. Short-term interest rate (Interest), which is measured by 3-month US treasury bill rate and obtained from the Federal Reserve Board’s H.10.
2. Exchange rate (Exch.), which is measured by the major currencies index collected from Federal Reserve Banks and obtained from the Federal Reserve Board’s H.15.
3. Inflation (Inflation), which is measured by the Consumer Price Index (CPI) and obtained from CRSP database.
4. Slope of the yield curve (Slope), which is measured by the spread between 10-year treasury rate and 3-month treasury rate and obtained from CRSP database.
5. Default rate (Default), which is measured by the difference between Moody’s Baa and Aaa corporate bond yields of the same maturity and obtained from Federal Reserve Board data files in WRDS database.
6. Consumer confidence (CC), which is measured by the Michigan Consumer Sentiment Index and obtained from Trading Economics.
7. Growth rate in the Industrial Production Index (IPI), which is obtained from Federal Reserve Board data files in WRDS database.
8. Unemployment rate (Unempl.), which is obtained from Trading Economics.

Utilizing the method in [28], we conduct the Principle Components Analysis (PCA) to these eight variables and select the first two principle components as macro-economic factors used in our model.

### Descriptive analysis

Tables 2 and 3 present the descriptive statistics for daily returns and 5-min returns of 29 DJIA stocks and S&P index, respectively.

**Table 2:** Descriptive statistics for daily returns.

Stock	Mean × 1000	25th Percentile × 1000	75th Percentile × 1000	SD × 1000	Skewness	Kurtosis
AA	-0.8021	-14.7440	15.0712	33.2524	-0.2453	5.8662
AXP	-0.0798	-11.0745	11.5691	29.8404	0.1049	6.4575
BA	0.0286	-10.2271	10.7482	20.7952	0.1065	3.6824
BAC	-1.3867	-13.5525	10.9586	43.5349	-0.2921	13.7690
CAT	0.3155	-11.6219	12.8381	24.0225	-0.0728	3.5591
CSCO	0.0425	-9.8890	10.7245	21.5366	-0.2270	6.6922
CVX	0.4070	-8.9778	10.5710	19.6762	0.1361	11.2978
DD	0.0494	-10.1948	10.6622	20.7331	-0.4140	4.6500
DIS	0.3111	-9.2823	9.6864	19.9378	0.3029	5.8237
GE	-0.4444	-8.9738	8.8330	23.1800	-0.1263	6.7691
HD	0.0271	-10.1650	10.0027	20.0926	0.3543	3.3508
HPQ	0.0150	-9.4746	10.4959	20.4492	0.0518	4.1362
IBM	0.5299	-6.5984	8.3069	15.1777	0.0212	4.2217
INTC	-0.0186	-10.7499	11.1148	21.0361	-0.1867	3.7357
JNJ	0.0490	-4.7435	5.2749	10.9609	0.1181	10.1205
JPM	-0.1124	-12.3190	11.2466	32.5366	0.3622	9.9782
KFT	0.1963	-6.4856	7.1668	13.5095	0.0097	3.8798
KO	0.3546	-5.5730	6.0818	12.7197	0.4636	8.5198
MCD	0.7355	-6.5178	7.8164	13.4517	-0.0439	3.8460
MMM	0.0280	-6.8518	8.0256	16.3772	-0.3299	4.7732
MRK	0.1455	-8.4768	9.4536	18.6671	-0.2957	6.2243
MSFT	-0.0063	-8.6208	8.7621	18.9069	0.1018	8.1691
PFE	-0.0239	-7.9898	8.3449	16.1736	-0.0495	4.5433
PG	0.0897	-4.8103	5.5626	12.1929	-0.1406	7.2973
T	0.1492	-7.7032	7.9086	16.2232	0.5071	8.2793
UTX	0.1827	-7.6566	8.8274	17.4147	0.2696	5.8673
VZ	0.1781	-7.0986	8.0542	15.8020	0.2347	7.0804
WMT	0.2052	-6.7874	6.5878	13.1521	0.1291	5.5092
XOM	0.2658	-8.1494	9.2605	18.2490	-0.0163	13.0925
S&P 500	0.0485	-5.4778	6.4014	15.3059	-0.1909	7.4016

**Table 3:** Descriptive statistics for 5-min returns.

Stock	Mean × 1000	25th Percentile × 1000	75th Percentile × 1000	SD × 1000	Skewness	Kurtosis
AA	-0.0170	-1.1251	1.1036	2.6924	-0.0922	15.9145
AXP	0.0135	-0.8775	0.8809	2.5181	0.2917	22.9642
BA	-0.0011	-0.7457	0.7478	1.7395	-0.2040	21.3570
BAC	-0.0240	-0.9307	0.8824	3.2044	-0.1366	32.5194
CAT	0.0009	-0.9121	0.9187	2.1086	0.2842	12.7307
CSCO	-0.0089	-0.8504	0.8252	1.8720	0.1516	15.1925
CVX	0.0011	-0.7753	0.7882	1.7979	-0.0055	20.2444
DD	-0.0002	-0.8005	0.8066	1.8509	-0.2325	25.5643
DIS	0.0083	-0.7163	0.7307	1.7045	-0.0389	23.6222
GE	-0.0041	-0.7620	0.7504	2.1056	0.4918	28.2999
HD	0.0017	-0.8085	0.7973	1.8943	0.1044	13.9980
HPQ	0.0027	-0.7484	0.7581	1.7736	1.1672	82.0893
IBM	0.0048	-0.5913	0.6060	1.4757	-0.6339	44.7640
INTC	-0.0037	-0.8559	0.8544	1.8677	-0.0364	24.9960
JNJ	0.0022	-0.4437	0.4413	1.0765	-0.4679	49.5585
JPM	0.0002	-0.9356	0.9266	2.6568	0.0829	23.6656
KFT	0.0061	-0.5369	0.5497	1.2937	0.0724	22.4096
KO	0.0020	-0.4996	0.5029	1.1862	-0.0108	22.3711
MCD	0.0034	-0.5607	0.5792	1.3207	-0.1335	21.7493
MMM	0.0015	-0.6413	0.6412	1.4869	-0.0982	22.6983
MRK	-0.0012	-0.7006	0.7023	1.6773	0.1270	49.9546
MSFT	-0.0036	-0.7170	0.7134	1.6315	-0.0946	13.9688
PFE	-0.0052	-0.6988	0.6874	1.5323	-0.0156	16.3319
PG	0.0008	-0.5052	0.5132	1.2062	-0.5129	50.5912
T	-0.0004	-0.6514	0.6497	1.6550	-0.4433	32.2104
UTX	0.0046	-0.6504	0.6588	1.5672	-0.2740	37.8865
VZ	0.0064	-0.6205	0.6364	1.5476	0.0266	27.5602
WMT	-0.0010	-0.5837	0.5779	1.3347	0.5412	30.5054
XOM	0.0003	-0.7262	0.7444	1.6712	-0.1574	25.4161
S&P 500	0.0009	-0.5111	0.5183	1.3497	0.0440	22.5620

Although the mean of daily returns and 5-min returns for S&P 500 index are approximately zero, their standard deviations are significantly different. It indicates that when sampling frequency increase, there is a dramatic change in volatility. Moreover, the skewness switches from -0.1909 for daily return to 0.0440 for 5-min return, which is more right-skewed. The kurtosis increases from 7.4016 for daily return to 22.5620 for 5-min return.

The standard deviations of individual stocks obtained from daily returns are significantly larger than those obtained from 5-min returns. Among the 29 individual stocks, 13 stocks have left-skewed distributions for daily return and 18 stocks have left-skewed distributions for 5-min return. Furthermore, the 29 stocks have larger kurtosis for 5-min return. The distributions of individual stock return are unsymmetrical with the long tail and sharp peak.

Table 4 further displays the correlation coefficients of eight macro-economic factors. It reveals the strong correlations between short-term interest rate and slope of the yield curve, between exchange rate and default rate, between growth rate in the Industrial Production Index and unemployment rate. Introducing all macroeconomic variables in covariance estimation model at one time will lead to a lack of statistical significance due to the presence of multicollinearity. Thus, we use principal component analysis (PCA) to create new independent variables and estimate inverse covariance matrix based on principle components in empirical study.

**Table 4:** Correlation coefficients between macro-economic variables.

	Interest	Exch.	Inflation	Slope	Default	CC	IPI	Unempl.
Interest	1							
Exch.	0.056	1						
Inflation	0.397	-0.309	1					
Slope	-0.521	-0.039	-0.333	1				
Default	-0.316	0.512	-0.371	0.163	1			
CC	-0.074	-0.077	-0.092	-0.040	-0.171	1		
IPI	0.175	-0.046	0.182	-0.059	-0.025	-0.317	1	
Unempl.	-0.053	0.157	-0.142	0.003	0.087	-0.010	-0.417	1

Table 5 shows the principle component matrix using PCA. The first principal component and second principal component we use explain 96.4945% of the total variance. The first component has significantly correlations with short-term interest rate and slope of the yield curve, which is called monetary factor. It is viewed as an indicator of monetary policy and bond market. The second component named as economic factor has positive correlations with default rate and exchange rate, which mostly captures the periodic fluctuations in economic activity.

**Table 5:** Principle component matrix.

Macro-economic variables	Principle component 1	Principle component 2
IPI	0.0016	0.0007
Default	-0.0361	0.1003
Exch.	0.0006	0.0078
Inflation	0.0019	-0.0011
Interest	0.4148	0.0085
Slope	-0.0257	-0.0002
Unempl.	-0.0016	0.0024
CC	-0.0046	-0.0212

## Empirical study

In our model, excess returns, which could be daily returns or 5-min returns, are driven by two daily observable factors: (i) the value-weighted stock market index; (ii) the innovation of the VIX index. The VIX index is constructed by the implied volatilities of S&P 500 index options. It indicates the investors’ expectation for future 30-day volatility of S&P500 index. We also include two monthly observable principle components of the eight macro-economic factors. We fit the proposed model, estimate the inverse covariance matrix, $S_{t,m,FMP}^{inv}$, and compute the minimum variance index tracking strategy with a rolling window scheme. And then we apply the derived strategy for the next day.

### Comparison of current different estimations

Here, we choose a rolling window of one year (252 trading days). Table 6 compares out-of-sample performances of minimum variance index tracking strategies, which are derived according to different covariance matrix or inverse covariance matrix estimators based on daily return, intraday return with overnight return and intraday return without overnight return, respectivley.

**Table 6:** Out-of-sample performances of minimum tracking error portfolios (T = 1 y).

Panel A: daily return					
Covariance	SD × 100	One-tail t test	Turnover (%)	25th Turnover (%)	75th Turnover (%)
***S***_*t*,1_	4.3973	-0.7453	3.3174	1.6897	4.2155
***S***_*t*,2_	4.9495	-3.2205	6.6824	3.7051	8.2612
***S***_*t*,3_	4.3569	0.5755	1.5611	0.7266	1.9814
$S_{t,4}^{inv}$	4.3973	-0.7453	3.3174	1.6897	4.2155
$S_{t,5}^{inv}$	4.2786	0.9673	2.5055	1.2826	3.1920
$S_{t,6}^{inv}$	4.3805	-0.5302	3.2310	1.6396	4.1386
$S_{t,7}^{inv}$	4.3837	0.1312	1.4594	0.7839	1.7540
$S_{t,8}^{inv}$	4.3943	-0.6026	3.3065	1.6867	4.2125
$S_{t,9}^{inv}$	4.3483	-0.4707	3.4507	1.8897	4.3523
Panel B: intraday return (with overnight)					
Covariance	SD × 100	One-tail t test	Turnover(%)	25th Turnover (%)	75th Turnover (%)
***S***_*t*,1_	4.5334	-1.4928	1.9859	1.1308	2.2552
***S***_*t*,2_	4.4037	1.5562	2.1551	1.2385	2.4591
***S***_*t*,3_	4.5944	-2.5103	1.1331	0.6547	1.2697
$S_{t,4}^{inv}$	4.5334	-1.4928	1.9859	1.1308	2.2552
$S_{t,5}^{inv}$	4.4053	0.6865	1.1572	0.7627	1.3270
$S_{t,6}^{inv}$	4.4553	-0.7483	1.7850	1.0500	2.0424
$S_{t,7}^{inv}$	4.5583	1.0620	0.9477	0.6554	1.0710
$S_{t,8}^{inv}$	4.4782	-0.6905	1.8969	1.0914	2.1464
$S_{t,9}^{inv}$	4.5042	-2.0110	1.9724	1.1696	2.3179
Panel C: intraday return (without overnight)					
Covariance	SD × 100	One-tail t test	Turnover(%)	25th Turnover (%)	75th Turnover (%)
***S***_*t*,1_	4.7817	-3.8486	1.8787	1.1561	2.0094
***S***_*t*,2_	4.4680	0.1306	2.2587	1.3622	2.4046
***S***_*t*,3_	4.7988	-3.1698	1.1020	0.6804	1.2085
$S_{t,4}^{inv}$	4.7817	-3.8486	1.8787	1.1561	2.0094
$S_{t,5}^{inv}$	4.4603	0.3487	1.0710	0.7222	1.1658
$S_{t,6}^{inv}$	4.6069	-2.9174	1.6364	1.0127	1.7634
$S_{t,7}^{inv}$	4.5661	0.4126	0.9483	0.6399	1.0783
$S_{t,8}^{inv}$	4.6563	-3.3159	1.7443	1.0761	1.8579
$S_{t,9}^{inv}$	4.7317	-4.1228	1.8818	1.1474	2.1217

Notes: standard deviations are annualized.

Several conclusions can be obtained from Table 6. First, tracking error achieved by ***S***_*t*,1_ with daily return is 0.043973, which is smaller than the tracking errors of using intraday return. Second, the shrinkage inverse covariance matrix estimator, $S_{t,5}^{inv}$, has the best out-of-sample performance (i.e., smallest tracking error). Third, when including intraday returns, introducing overnight returns often yields better tracking performance, which implies that the overnight returns are useful. Fourth, compared with covariance matrix estimators, most inverse covariance matrix estimators do not have significantly better performances. Furthermore, Table 6 provides turnover rates of applying different strategies. The turnover rates of strategies based on intraday return are smaller, which indicates the value of intraday returns. The tracking strategy with the estimator $S_{t,7}^{inv}$ has the lowest turnover rate.

Surprisingly, involving intraday returns may lower the tracking performance but improve the turnover rates for these estimators. What’s more, overnight returns are meaningful to improve the tracking performance.

### Performance of FACTOR-MIDAS-POET method

We consider two models. The first one is based on daily returns of stocks, two daily observable factors, and two monthly macro-economic principle components. The second one is based on 5-min returns of stocks, two daily observable factors, and two monthly macro-economic principle components. Similar to [30], we estimate the models with slow weights (*θ*_1_ = 1 and *θ*_2_ = 4). We also try other forms of weights and obtain the similar conclusions.

Table 7 presents out-of-sample tracking performances of strategies based on FACTOR-MIDAS-POET model. The lag period column reports how many monthly macro-economic principle components are included in the models. Panel A presents the results based on daily returns, while Panel B and Panel C present the results based on 5-min returns. We report the one-tailed t test of tracking errors of different portfolios based on Newey-West standard deviations with six lags in Tables 6 and 7. Following [39], we examine the average squared excess returns over the target index of various estimates with the average squared excess returns of FACTOR-MIDAS-POET method (*K* = 3). To test the average of $S_{1,F-M-P}^{2}$, $S_{2,F-M-P}^{2}$, …, $S_{N,F-M-P}^{2}$ is significantly smaller than the average of $S_{1,others}^{2}$, $S_{2,others}^{2}$, …, $S_{N,others}^{2}$, we test whether the mean of the sequence $\text{log}(S_{1,F-M-P}^{2}/S_{1,others}^{2})$, $\text{log}(S_{2,F-M-P}^{2}/S_{2,others}^{2})$, …, $\text{log}(S_{N,F-M-P}^{2}/S_{N,others}^{2})$ is significantly smaller than zero using a one-tailed test.

**Table 7:** Out-of-sample performances of FACTOR-MIDAS-POET method (T = 1 y).

Panel A: daily return					
Lag period (K)	SD × 100	One-tail t test	Turnover (%)	25th Turnover (%)	75th Turnover (%)
3 months	4.3068		2.5313	1.3383	3.1822
6 months	4.3037	0.5512	2.5374	1.3373	3.1671
9 months	4.3003	1.5556	2.5259	1.3181	3.1328
1 year	4.2983	1.5215	2.5357	1.3237	3.1416
Panel B: intraday return (with overnight)					
Lag period (K)	SD × 100	One-tail t test	Turnover (%)	25th Turnover (%)	75th Turnover (%)
3 months	4.3492		7.9411	3.8282	10.6202
6 months	4.3732	0.8224	7.4164	3.2755	9.7167
9 months	4.3968	0.8798	7.7529	2.5058	10.4505
1 year	4.4043	-0.4362	7.7152	2.6545	10.2169
Panel C: intraday return (without overnight)					
Lag period (K)	SD × 100	One-tail t test	Turnover (%)	25th Turnover (%)	75th Turnover (%)
3 months	4.3711		7.5717	2.6967	10.5070
6 months	4.3968	0.5092	7.1411	2.7568	9.6251
9 months	4.4179	0.0177	7.8259	2.7595	10.4848
1 year	4.4240	-0.2833	7.8535	2.8240	10.5540

Some conclusions can be obtained from Table 7. First, the tracking performance and turnover ratio based on daily returns are both better than those based on 5-min returns. Second, the tracking performance of our model depends on how many monthly macro-economic principle components are included. Third, except ***S***_*t*,3_, $S_{t,5}^{inv}$ and $S_{t,7}^{inv}$, the tracking performance and turnover ratio of proposed model are both better than other covariance estimation models based on daily returns. Meanwhile, the one-tail t value of ***S***_*t*,3_, $S_{t,5}^{inv}$ and $S_{t,7}^{inv}$ are not great. Similarly, compared with most models reported in Table 6, the proposed FACTOR-MIDAS-POET model has better out-of-sample tracking performance using intraday return. Because our method utilizes financial data with different resources and frequencies. However, the turnover rate of our model is high. This indicates that investment strategies constructed by our method are more active. The high turnover rate is not always a negative indicator. When trying to minimize the index tracking errors for investors, the index investment strategy constructed by our model can reach the targeted performance.

### Robust analysis

In this robust analysis, we change the rolling window into three months (3 m), six months (6 m) and nine months (9 m), in order to check whether the length of rolling windows affects our main results.

Tables 8–10 summarize out-of-sample performances of strategies based on different models under three different lengths of rolling windows. When the length of estimation window decreases, tracking errors and turnover rates of all models increase. Because less information is used. Meanwhile, as the length of estimation window decreases, the value of 5-min return data becomes more and more important. Therefore, the intraday information can help us estimate covariance matrix or inverse covariance matrix and construct investment strategies when there is a lack of other information. Most importantly, the proposed FACTOR-MIDAD-POET model has better tracking performance than other models in general, especially when the rolling window is three months. Thus, when the length of estimation window is relatively short, introducing macro-economic information and option market information can greatly improve index tracking strategy.

**Table 8:** Out-of-sample performances of portfolios with T = 9 m.

	daily return			intraday return (with overnight)			intraday return (without overnight)		
	SD × 100	One-tail t test	Turnover (%)	SD × 100	One-tail t test	Turnover (%)	SD × 100	One-tail t test	Turnover (%)
***S***_*t*,1_	4.3962	-0.7695	4.3867	4.5215	-2.5842	2.4893	4.8109	-3.8418	2.2793
***S***_*t*,2_	5.0352	-4.2256	9.6052	4.3443	0.8050	2.7081	4.4254	-0.2626	2.8732
***S***_*t*,3_	4.3598	0.2221	1.5679	4.5997	-3.0435	1.1360	4.7988	-3.5596	1.1020
$S_{t,4}^{inv}$	4.3962	-0.7695	4.3867	4.5215	-2.5842	2.4893	4.8109	-3.8418	2.2793
$S_{t,5}^{inv}$	4.2696	0.9205	3.1846	4.3977	-0.2501	1.2425	4.4610	-0.6451	1.1080
$S_{t,6}^{inv}$	4.3725	-0.4960	4.2268	4.4134	-1.4729	2.1106	4.5698	-3.4845	1.8424
$S_{t,7}^{inv}$	4.3613	-0.1750	1.8272	4.5454	0.4413	0.9602	4.5559	-0.4769	0.9560
$S_{t,8}^{inv}$	4.3921	-0.2910	4.3656	4.4424	-1.5873	2.3111	4.6302	-3.6113	2.0235
$S_{t,9}^{inv}$	4.3237	-0.3848	4.6659	4.4805	-3.4180	2.4042	4.7448	-4.0267	2.2522
FACTOR-MIDAS-POET Method									
K = 3 m	4.3079		2.9523	4.3034		7.6426	4.3709		7.1357
K = 6 m	4.3172	1.3872	2.9439	4.2937	-0.3152	9.3044	4.3606	1.2395	9.0471
K = 9 m	4.3202	1.2288	2.9333	4.2861	0.5328	10.2419	4.3515	-0.3981	10.2960
K = 1 y	4.3181	0.7466	2.9268	4.2861	1.8189	10.5034	4.3491	-0.3919	10.8934

**Table 9:** Out-of-sample performances of portfolios with T = 6 m.

	daily return			intraday return (with overnight)			intraday return (without overnight)		
	SD × 100	One-tail t test	Turnover (%)	SD × 100	One-tail t test	Turnover (%)	SD × 100	One-tail t test	Turnover (%)
***S***_*t*,1_	4.5932	-1.5837	7.1564	4.5772	-1.3815	3.4341	4.9135	-3.0659	2.9827
***S***_*t*,2_	5.6487	-5.9653	17.1580	4.3574	0.0090	3.7923	4.5930	-0.9154	4.1070
***S***_*t*,3_	4.3676	0.3467	1.5805	4.6044	-2.5855	1.1412	4.7986	-2.8346	1.1020
$S_{t,4}^{inv}$	4.5932	-1.5837	7.1564	4.5772	-1.3815	3.4341	4.9135	-3.0659	2.9827
$S_{t,5}^{inv}$	4.3597	-0.5670	4.8490	4.4063	1.0346	1.3557	4.4801	0.8104	1.1356
$S_{t,6}^{inv}$	4.5404	-1.7153	6.7657	4.3990	-0.3520	2.6126	4.5555	-1.5450	2.0895
$S_{t,7}^{inv}$	4.3750	1.1381	2.9238	4.5292	0.2777	0.9860	4.5485	0.0680	0.9668
$S_{t,8}^{inv}$	4.5829	-2.1251	7.1017	4.4431	-0.5275	3.0179	4.6282	-2.0229	2.4208
$S_{t,9}^{inv}$	4.4795	-0.4644	7.6952	4.5050	-1.6695	3.2569	4.8155	-3.2856	3.0981
FACTOR-MIDAS-POET Method									
K = 3 m	4.3536		4.1701	4.2871		13.4000	4.3858		13.8514
K = 6 m	4.3482	1.3738	4.2596	4.3092	0.3213	12.9060	4.4069	0.1706	13.3304
K = 9 m	4.3475	1.5734	4.1916	4.3106	1.1315	12.5355	4.4064	0.0551	13.0920
K = 1 y	4.3467	1.6594	4.2397	4.3075	0.8631	12.2162	4.4041	-0.0793	12.8645

**Table 10:** Out-of-sample performances of portfolios with T = 3 m.

	daily return			intraday return (with overnight)			intraday return (without overnight)		
	SD × 100	One-tail t test	Turnover (%)	SD × 100	One-tail t test	Turnover (%)	SD × 100	One-tail t test	Turnover (%)
***S***_*t*,2_	7.5510	-9.9487	54.5114	4.7384	-0.1167	9.3569	5.1382	0.2119	10.7838
***S***_*t*,3_	4.3720	1.3574	1.6026	4.6092	-2.6760	1.1474	4.7990	-3.1201	1.1023
$S_{t,4}^{inv}$	5.7823	-4.8004	24.0316	4.7317	-1.4858	6.9206	4.9736	-3.0404	5.5949
$S_{t,5}^{inv}$	4.6812	-1.1423	13.8312	4.4119	1.1202	1.4502	4.4645	-0.1240	1.1311
$S_{t,6}^{inv}$	5.4748	-3.7443	21.6904	4.2959	2.1670	3.4687	4.3263	1.3167	2.3255
$S_{t,7}^{inv}$	4.6957	-0.6223	13.1463	4.3981	0.6004	1.1569	4.4206	-0.7299	1.0495
$S_{t,8}^{inv}$	5.7178	-4.4180	23.6444	4.3432	1.8964	4.7818	4.3408	0.8790	3.0793
$S_{t,9}^{inv}$	5.2228	-3.1534	24.9688	4.5047	-0.6196	6.0299	4.7502	-3.4748	5.3643
FACTOR-MIDAS-POET Method									
K = 3 m	4.5092		7.5565	4.3852		14.8179	4.4330		14.2007
K = 6 m	4.5023	0.0629	7.6250	4.3960	-0.1793	14.8875	4.4467	-0.3520	13.9836
K = 9 m	4.4996	-0.3466	7.6462	4.4041	-0.9606	14.6158	4.4541	-0.6767	13.7511
K = 1 y	4.4993	-0.7947	7.6341	4.4032	-0.7387	14.0725	4.4540	-0.6693	13.1841

## Conclusions and discussion

In this paper, we propose the FACTOR-MIDAS-POET model, which integrates the intraday return data, daily risk factors data and monthly or quarterly macro economy data, simultaneously. In empirical analysis, we show that the FACTOR-MIDAS-POET model with macro-economic factors has better out-of-sample tracking performance than most current used models in the literature. The proposed model can fully utilize the financial data with different resources and frequencies. However, the better tracking performance often accompanies with higher turnover rates. Meanwhile, we find that our model has better performance as the length of the estimation window decreases.

Our work is a preliminarily study on the index tracking with mix-frequency data. There are still a lot of aspects we do not cover. For instance, we do not consider the noise of intraday data in the model. Also, when studying index tracking problems, the transaction cost is not explicitly appeared in the model. These problems are worth to be further explored in future studies.

## Supporting information

S1 Dataset (XLSX)
